# Molecular Variation and Phylogeny of Thymidylate Kinase Genes of *Candidatus* Phytoplasma ziziphi from Different Resistant and Susceptible Jujube Cultivars in China

**DOI:** 10.3390/biology13110886

**Published:** 2024-10-30

**Authors:** Chuan-Sheng Song, Qi-Cong Xu, Cui-Ping Wan, De-Zhi Kong, Cai-Li Lin, Shao-Shuai Yu

**Affiliations:** 1 College of Agricultural and Biological Engineering, Heze University, Heze 274015, China; cssonghy@163.com (C.-S.S.); wcp423@163.com (C.-P.W.); 2 International Nature Farming Research Center, Nagano 390-1401, Japan; qicongx@163.com; 3 Key Laboratory of Forest Protection of National Forestry and Grassland Administration, Ecology and Nature Conservation Institute, Chinese Academy of Forestry, Beijing 100091, China; kongdz@caf.ac.cn; 4 Coconut Research Institute, Chinese Academy of Tropical Agricultural Sciences, Wenchang 571339, China

**Keywords:** phytoplasma, host plant resistance, thymidylate kinase gene, evolutionary pathway, potential mobile unit

## Abstract

The thymidylate kinase (*tmk*) gene is indispensable for the proliferation and survival of phytoplasma. To reveal the molecular variation and phylogeny of the *tmk* genes of *Candidatus* phytoplasma ziziphi, in this study, the *tmk* genes of 50 phytoplasma strains infecting different resistant and susceptible jujube cultivars from different regions in China were amplified and analyzed. Two sequence types, *tmk-x* and *tmk-y*, were identified using clone-based sequencing. The JWB phytoplasma strains were classified into three types, type-X, type-Y, and type-XY, based on the sequencing chromatograms of the *tmk* genes. The type-X and type-Y strains contained only *tmk-x* and *tmk-y* genes, respectively. The type-XY strain contained both *tmk-x* and *tmk-y* genes. The type-X, type-Y, and type-XY strains comprised 42%, 12%, and 46% of all the strains, respectively. The type-X and type-XY strains were identified in both susceptible and resistant jujube cultivars, while type-Y strain was only identified in susceptible cultivars. Phylogenetic analysis indicated that the *tmk* genes of the phytoplasmas were divided into two categories: phylo-S and phylo-M. The phylo-S *tmk* gene was single-copied in the genome, with an evolutionary pattern similar to the 16S rRNA gene; the phylo-M *tmk* gene was multi-copied, related to PMU-mediated within-genome transposition and between-genome transfer. Furthermore, the phylogenetic tree suggested that the *tmk* genes shuttled between the genomes of the Paulownia witches’ broom phytoplasma and JWB phytoplasma. These findings provide insights into the evolutionary and adaptive mechanisms of phytoplasmas.

## Simple Summary

Phytoplasmas are phloem-limited bacteria that cannot be cultured in vitro. The thymidylate kinase (*tmk*) genes of 50 phytoplasma strains infecting jujube cultivars with different resistances in China were amplified and analyzed. Two sequence types, *tmk-x* and *tmk-y*, were identified, and the 50 JWB phytoplasma strains were classified into three types, type-X, type-Y, and type-XY, based on the *tmk* sequence types. The type-X, type-Y, and type-XY strains comprised 42%, 12%, and 46% of all the strains, respectively. The strains with *tmk-x* could be identified in susceptible and resistant jujube cultivars, while the strains just with *tmk-y* were only identified in susceptible cultivars. Phylogenetic analysis showed that the *tmk* genes of the phytoplasmas were divided into two clades. The *tmk* genes in one clade were single-copied in the genome with an evolutionary pattern similar to the 16S rRNA gene; the genes in the other clade were multi-copied related to PMU-mediated within-genome transposition and between-genome transfer. This study will benefit further understanding the genetic variations and the mechanisms behind phytoplasma adaptive evolution.

## 1. Introduction

Chinese jujube (*Ziziphus jujuba* Mill), indigenous to China, has a long cultivation history and is widely distributed across the country, with important economic values [1]. Jujube witches’ broom (JWB), related to a phytoplasma, is a systemic infectious disease characterized by abnormal branching, malformed floral organs, small leaves, and yellowing, and typically results in death within 2–3 years after the infection [2]. This disease is almost distributed in all jujube cultivation areas in China, causing a major disaster to jujube producers [3].

Phytoplasma is an unculturable bacterium. It specially parasitizes in the phloem sieve tubes of host plants or in the salivary glands and intestines of vector insects [2]. In order to survive, bacteria undergo variations to adapt to changing environments [4]. The modulation of bacterial proliferation is an important survival strategy [5]. The *tmk* gene is a crucial proliferation-related gene that is present in almost all organisms [6]. It is associated with DNA synthesis and repair [7]. The TMK protein encoded by this gene catalyzes the transfer of a terminal phosphoryl group from ATP to dTMP and is crucial to both the de novo synthetic and salvage pathways for pyrimidine deoxyribonucleotides [6]. The absence of a *tmk* gene may lead to organisms’ inability to survive. Therefore, it is an important gene for studying the adaptive evolution of phytoplasma.

The adaptive evolution of phytoplasma is potentially related to PMU, a unique potential mobile element specific to phytoplasma, which contains *tmk* and several other genes [8,9]. The *tmk* gene is multi-copied in almost all sequenced phytoplasma genomes. For instance, a complete genome of the PaWB-Zhengzhou phytoplasma from China contains eleven *tmk* copies, six of which differ [10]. There are four and five *tmk* gene copies in two JWB phytoplasma complete genomes, JWB-Hebei-2018 [11] and JWB-nky [12], respectively, from China. Four *tmk* copies of JWB-Hebei-2018 are distinct from each other [11], and there are two different sequences among the five *tmk* copies of JWB-nky [12]. Due to the widespread distribution of JWB diseases, China has a rich variety of JWB phytoplasma strains. However, except for JWB-nky and JWB-Hebei-2018 strains, the *tmk* gene sequences of JWB phytoplasma strains remains unknown in China. Although there have been a few experimental studies on the variations of other phytoplasma *tmk* genes [13,14,15], they have not revealed the effects of variations in adaptability.

There are comparatively abundant germplasm resources of jujube trees in China, with more than 700 cultivated varieties [1]. The resistance level of many jujube cultivars to JWB phytoplasma has been identified [16,17,18,19]. Since *tmk* is an essential survival-related gene, revealing the relationship between the variation of the *tmk* gene in JWB phytoplasma and the resistance of jujube cultivars is necessary for elucidating the mechanism of phytoplasma adaptation to the host. Furthermore, clarifying the phylogenetic relationships of the numerous and diverse known *tmk* genes could reveal its origins and revolutionary pathways.

In this study, JWB samples of different resistant varieties were collected from different cultivation areas in China. Two *tmk* gene sequences of these phytoplasma strains were identified. The variation and phylogeny of the *tmk* genes of the JWB phytoplasmas were analyzed. Then, we obtained some notable results and conclusions regarding the adaptation of the *tmk* gene variations to host plants, PMU-mediated *tmk* gene shuttling, and the classification and evolutionary pathways of the *tmk* gene of phytoplasmas. This study provides references for further clarifying the evolutionary and adaptive mechanisms of these pathogens.

## 2. Materials and Methods

### 2.1. Source of Materials and DNA Extraction

A total of 50 field samples of jujube cultivars infected with JWB phytoplasmas were collected from 8 representative jujube cultivation regions in China. Our previous studies confirmed that all these samples were only infected with JWB phytoplasmas [20]. Fresh shoots with typical witches’ broom were subjected to an immediate DNA extraction. The healthy control jujube was collected from a phytoplasma-free orchard in Jiangxi Province, China. Each sample was treated as an isolate (strain) of JWB phytoplasmas and assigned a unique abbreviation. The information on the JWB samples is listed in Table S1. Some JWB phytoplasma strains were also maintained on in vitro-cultured jujube plantlets in our laboratory. According to the manufacturer’s instructions (Aidlab Biotechnologies CO., LTD., Beijing, China), total DNA was extracted from the samples using the DNA Secure Plant Kit. To ensure accuracy and prevent contamination, a water control (without template DNA) was included in each PCR reaction.

### 2.2. PCR Amplification of Tmk Gene

The primer pairs tmka-N (5′-TTGAATTCCATATGAAATTAATCGTTTTTGAAG GACT-3′)/tmka-C (5′-TGAGCTCGAGTTAGTTATGATCGCCATTTGATAGTACT-3′) and tmkb-N (5′-TTGAATTCCATATGTTTATTTCTTTTGAAGGTTGTGA-3′)/tmkb-C (5′-TGA GCTCGAGCTATTTGAAAGACTTCTTTGAGTTTTGT-3′) reported by Miyata [13] were used to amplify the *tmk* genes. PCR amplification was performed in 25 μL volumes containing 0.5 μL (10 μM) of each primer, 12.5 μL of 2 × Taq PCR Mix (Tiangen Biotech Beijing Co., Ltd., Beijing, China), 10.5 μL of ddH_2_O, and 1 μL of the undiluted DNA preparation. The reaction conditions were as follows: an initial denaturation step at 94 °C for 4 min, followed by 35 cycles consisting of denaturation at 94 °C for 30 s, annealing at 48.5 °C for 30 s, and extension at 72 °C for 40 s, with a final extension at 72 °C for 5 min. The PCR products were visually detected using 1% (*w*/*v*) agarose gel electrophoresis with ethidium bromide staining.

### 2.3. Clone-Based and Direct Sequencing of Tmk Gene

The PCR products were purified using a DNA purification kit (TIANGEN Biotech (Beijing, China) Co., Ltd.), and then ligated into a pMD18-T simple vector. The ligation reaction was performed in 5 μL volumes containing 1 μL of the pMD18-T simple vector, 4 μL of purified *tmk* gene fragments, and 5 μL of solution I containing Ligase. The reaction was incubated at 16 °C for 1 h, transformed into *Escherichia coli* DH5α cells, and selected on LB agar plates containing 50 μg/mL ampicillin.

Two DNA-sequencing methods were employed: sequencing after the PCR products were cloned to the pMD18-T simple vector (Takara biotechnology (Dalian) Co., Ltd., Dalian, China) and the direct sequencing of the PCR products using an ABI PRISM 3730XL DNA sequencer [20]. The sequences were aligned using DNAMAN 7.0.

### 2.4. Selective PCR Amplification of Different Types of Tmk Gene

To identify the two *tmk* gene types in the JWB phytoplasma isolates, *tmk-x,* and *tmk-y*, the primer pair xtmkf1 (5′-GTG ATT TAT CTT AAA AAT TTG-3′)/xtmkr (5′-CTT CTC TTA TTC ACG CCC TTC AA-3′) was designed based on the *tmk-y* gene’s sequence to specifically amplify the *tmk-y* gene. The primer pair xtmkf2 (5′-ATG ATT TAT CTT AAA GAT CTT-3′/xtmkr (5′-CTT CTC TTA TTC ACG CCC TTC AA-3′) was designed according to the *tmk-x* gene’s sequence to specifically amplify the *tmk-x* gene. The amplification procedure and reaction system were the same as that of tmka-N/tmka-C [13].

### 2.5. Phylogenetic Analysis

Evolutionary analyses were conducted in MEGA 7.0 [21]. The evolutionary history was inferred using the neighbor-joining method [22]. The percentages of replicate trees in which the associated taxa clustered together in the bootstrap test (1000 replicates) were shown next to the branches [23]. The tree was drawn to scale, with branch lengths in the same units as those of the evolutionary distances used to infer the phylogenetic tree. The evolutionary distances were computed using the p-distance method [24] and were in the units of the number of base differences per site. We refined the phylogenetic tree using ChiPlot software (https://www.chiplot.online) [25].

## 3. Results

### 3.1. Two Sequence Types of Tmk Genes of JWB Phytoplasmas

**Figure 1:**
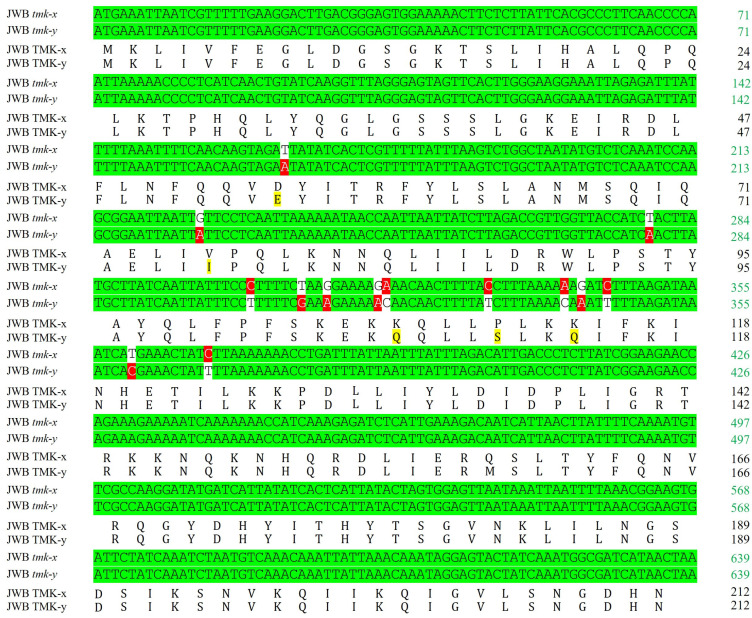
Variation sites of the *tmk* genes and amino acids of the coding proteins of the JWB phytoplasma. The *tmk-x* and *tmk-y* gene sequences, along with the amino acid sequences of their encoded proteins TMK-x and TMK-y, were aligned, respectively, using DNAMAN 7.0 software. The nucleotide sequence is highlighted with a green background. The mutated nucleotides are highlighted with a red background. The amino acid sequences are not highlighted. The mutated amino acids are highlighted with a yellow background.

A PCR product with a length of 640 bp was obtained using the primer pair tmk-a/tmka-C from all the diseased samples. No specific PCR product was obtained from the healthy control samples. No specific product was obtained using tmkb-N/tmkb-C [13]. In total, 181 clones of 16 strains were sequenced. Two sequence types of *tmk* genes were identified, namely, *tmk-x* and *tmk-y*, the sequences of which are deposited in the NCBI GenBank with the accession numbers GU196274 and GU196275. Open reading frame (ORF) predication, sequence alignment, and similarity analysis showed that the *tmk-x* and *tmk-y* genes had a complete ORF, contained 639 nucleotides, and encoded 212 amino acids. The *tmk-x* and *tmk*-*y* genes shared a 97.8% nucleotide identity, while their coding proteins shared a 97.6% amino acid identity. Fourteen consistent variable nucleotide sites were identified, resulting in five amino acid substitutions between the sequences (Figure 1).

### 3.2. Three Types of JWB Phytoplasma Strains Were Divided Based on the Tmk Genes

The sequencing chromatograms of the 50 strains listed in Table S1 were obtained by the sequencing of the direct PCR products. These strains were mainly divided into three types based on the sequencing chromatograms shown in Figure S1. The first type was ‘type-X’, containing 21 phytoplasma strains with the *tmk-x* gene sequence, which showed a single peak at 14 nucleotide positions. The second type was ‘type-Y’, containing six phytoplasma strains with the *tmk-y* gene sequence, which showed a single peak with different nucleotides at the above-matched positions. The third type was ‘type-XY’, containing 23 phytoplasma strains, which showed double peaks at the corresponding positions. One peak was identical to the type-X phytoplasma strains, and another was identical to type-Y phytoplasma strains. The type-XY strain may contain both *tmk-x* and *tmk-y* genes or could be a mixture of strains, including type-X and type-Y ones.

**Figure 2:**
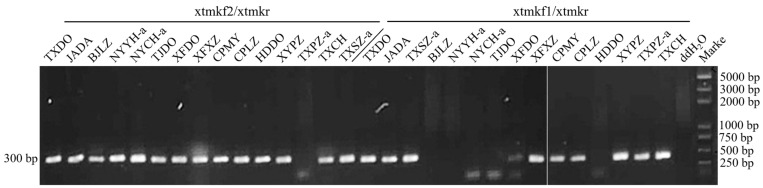
Selective PCR amplification of the different types of the JWB Phytoplasma strains. The 15 strains were amplified by the PCR using the specific primer pair xtmkkf2/xtmkr for the *tmk-x* gene and xtmkf1/xtmkr for the *tmk-y* gene. The abbreviation of each strain are shown in Table S1.

Selective PCR amplification based on the nucleotide differences between the *tmk-x* and *tmk-y* genes was performed to further confirm the existence of the three types of JWB phytoplasma strains. The PCR products of 15 representative strains were obtained (Figure 2; Table S1). The type-XY and type-X strains showed positive results when using the primer pair xtmkf2/xtmkr designed specifically for the *tmk-x* gene, while the type-Y strains showed negative results. The type-XY and type-Y strains showed positive results when using the primer pair xtmkf1/xtmkr designed specifically for the *tmk-y* gene, while the type-X strains showed negative results. These selective PCR amplification results all coincided with the results from the direct sequencing of the PCR products.

### 3.3. Structure and Functional Motifs of TMK Proteins

**Figure 3:**
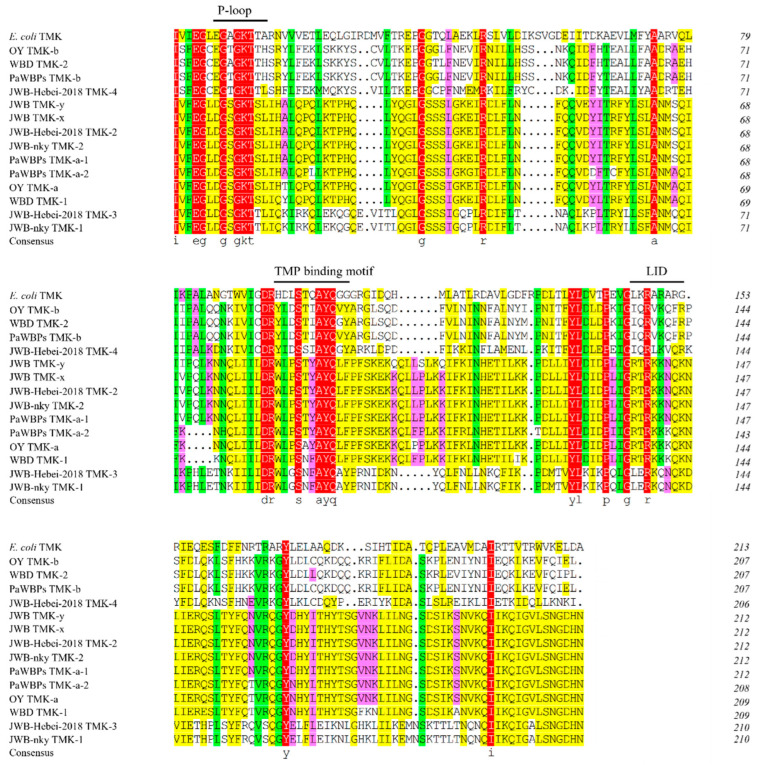
Multiple sequence alignment and functional domain analysis of TMK from different phytoplasmas. A total of 15 TMK amino acid sequences were aligned using DNAMAN 7.0 software. P-loop, TMK binding motif, and LID were functional domains of TMK proteins. Different colors represent varying levels of homology. Red, green, yellow and pink represent 100%, ≥70%, ≥50% and ≥33% identity respectively.

The TMKs of all organisms have three conserved specific motifs that are involved in NTP/NMP binding; the P-loop domain (N′-GXXGXGKT-C′, where X indicates an arbitrary residue), involved in the binding of ATP and other phosphoryl donors; the TMP-binding motif (N′-DRXXXSXXAYQ-C′), involved in the binding of nucleoside monophosphate; and the LID region (N′-XGXXRXXX-C′), which is a phosphoryl donor-binding site [13,14,15]. In this study, both the TMK-x and TMK-y of the JWB phytoplasmas contained all three motifs. The functional motifs of the TMK-x were identical to the TMK-y, including 8-GLDGSGKT-15, 88-DRWLPSTYAYQ-98, and 139-IGRTRKKN-146 (Figure 3). The degree of conservation of the LID was much lower than the P-loop domain and TMP-binding motif. These results reveal that the TMK-x and TMK-y of JWB phytoplasmas have conserved functional motifs, suggesting they may function as TMK enzymes.

### 3.4. Relationships Between Tmk Sequence Types and Jujube Cultivars with Different Resistance Levels from Different Geographic Distributions

**Figure 4:**
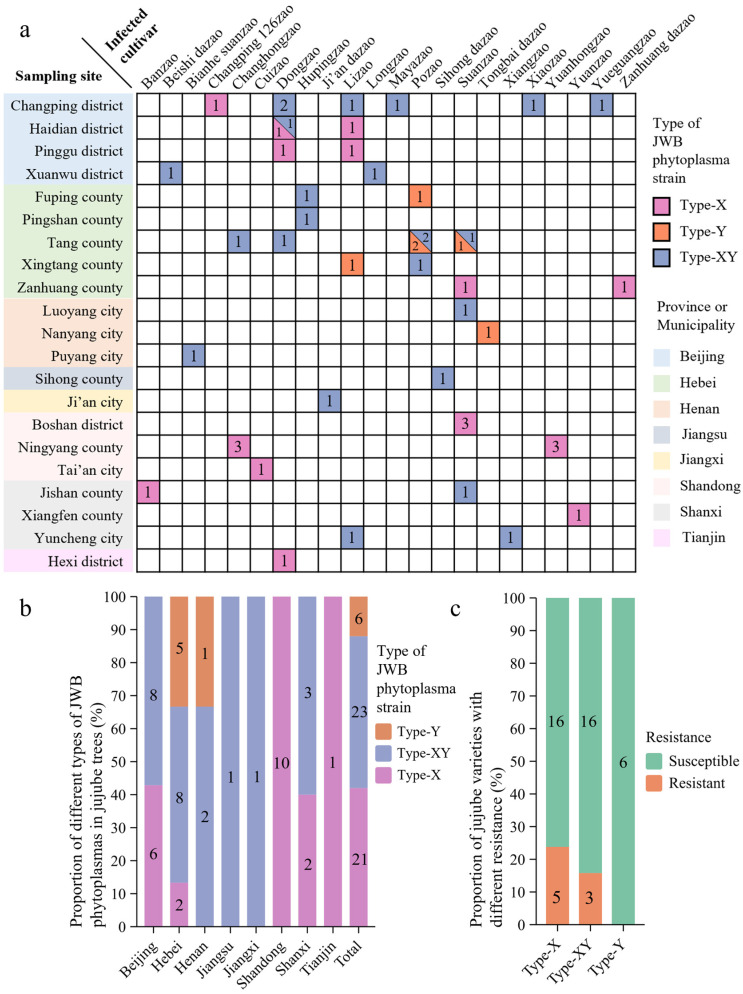
The type of phytoplasma strain is related to the resistance and geographic distribution of jujube cultivars. (**a**) The type data of the JWB phytoplasma strains in different regions and host varieties are visualized. The sampling sites of the infected cultivar and the types of JWB phytoplasma strains are shown in Table S1. (**b**) The proportion of the different types of JWB phytoplasmas of eight provinces or municipalities. (**c**) The proportion of resistant and susceptible hosts, respectively, infected with JWB phytoplasma strains with type-X, type-Y and type-XY. The numbers on the columns with different colors in (**a**,**b**) represent the number of JWB phytoplasma strains with different types. The numbers on the columns with different colors in (**c**) represent the number of JWB phytoplasma strains infecting the resistant and susceptible jujube cultivars.

The types of 50 JWB phytoplasma strains infecting jujube cultivars with different resistance levels from different regions in China are visualized and shown in Figure 4. Three types of phytoplasma strains, type-X, type-Y, and type-XY, comprised 42%, 12%, and 46% of all the strains, respectively. The type-X and type-XY phytoplasma strains were identified across eight sampled regions, whereas the type-Y phytoplasma strains were confined to the Hebei and Henan Provinces (Figure 4a,b and Table S1). These results indicate that type-X and type-XY were the dominant strains, while type-Y was the subordinate strain. Furthermore, the resistant and susceptible levels of the jujube cultivars used in this study were described in previous research [16,17,18,19], as indicated in Table S1. Of these, eight jujube trees were resistant to phytoplasmas, and the others were susceptible. Although all these jujube trees showed symptoms of witches’ broom, the severity of those of the eight resistant jujube trees was lower than those of the other susceptible jujube trees. Then, the numbers of resistant and susceptible jujube trees harboring the phytoplasma strains with type-X, type-XY, and type-Y were analyzed to explore the relationships among the types of the phytoplasma strains and the resistant levels of the jujube cultivars (Figure 4c). Among the jujube cultivars infected by the type-X or type-XY phytoplasma strains, some cultivars were susceptible (e.g., ‘Dongzao’, ‘Pozao’, ‘Lizao’, ‘Yuanhongzao’, and ‘Longzao’) and others were resistant (e.g., ‘Hupingzao’, ‘Banzao’, and ‘Changhongzao’). All the jujube cultivars infected by the type-Y phytoplasma strains were relatively susceptible. These findings suggest that type-X and type-XY phytoplasma strains might be capable of infecting resistant cultivars, while type-Y phytoplasma strains are restricted to susceptible cultivars.

### 3.5. Two Different Evolutionary Pathways of the Tmk Genes of Phytoplasmas Revealed via the Phylogenetic Tree

**Figure 5:**
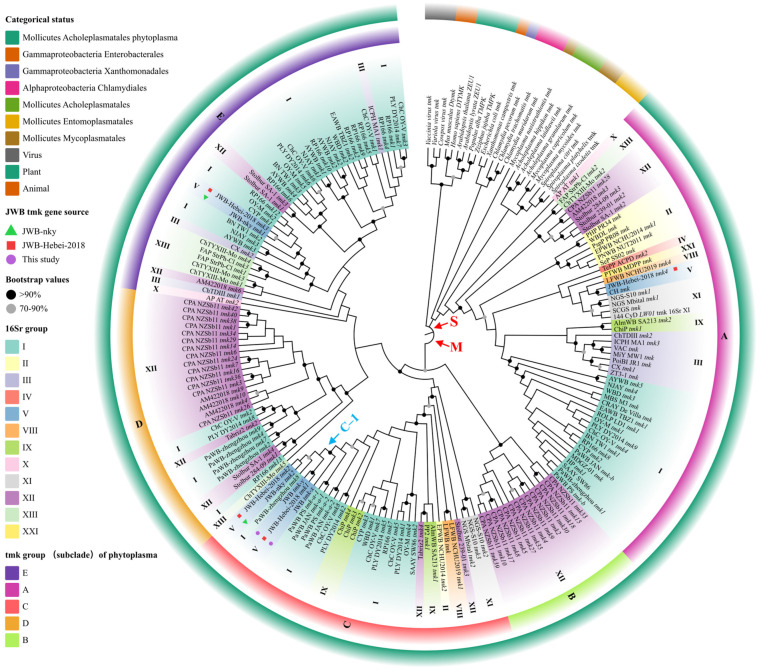
The phylogenetic tree of the *tmk* gene in phytoplasmas. The analysis involved 191 nucleotide sequences, as shown in Table S2. The evolutionary history was inferred using the neighbor-joining method. The percentages of the replicate trees in which the associated taxa clustered together in the bootstrap test (1000 replicates) are shown next to the branches. Black circles indicate the percentages of replicate trees greater than 90%, gray circles indicate percentages between 60 and 90%, and unmarked circles indicate percentages less than 60%.

The *tmk* gene sequences of the phytoplasmas deposited in GenBank were collected to clarify the evolutionary relationship. Only one among several identical *tmk* sequences from a phytoplasma strain was reserved. In total, 189 *tmk* sequences from GenBank (Table S2), together with two *tmk* gene sequences from this study, were aligned. Subsequently, the phylogenetic tree was constructed. Two ancestor clades, S and M, were featured in the phylogenetic tree, as shown in Figure 5. Accordingly, the *tmk* genes of the phytoplasmas worldwide were classified into two major categories: phylo-S *tmk* and phylo-M *tmk*. According to the screening in GenBank, most phytoplasmas contained above two categories (Table S2). Moreover, the phylo-S *tmk* was single-copied, and the phylo-M *tmk* was usually multi-copied in a phytoplasma genome. In total, 51 *tmk* genes from 51 different phytoplasmas were clustered into clade S, which also contained *tmk* genes from animals, plants, bacteria, and viruses. Furthermore, its genetic distance range within clade S was narrower than clade M (Table S3), suggesting that the phylo-S *tmk* was more conserved than the phylo-M *tmk*. In clade S, the *tmk* genes from the same 16S rDNA group were adjacent, and their evolutionary relationship was similar to the 16S rRNA gene (Figure S2), indicating that the phylo-S *tmk* had an evolutionary process like the 16S rRNA gene.

In clade M, *tmk* genes from a phytoplasma often could not be clustered together but were distributed in different subclades, which could be attributed to the multi-copy and diversity of phylo-M *tmk* genes. For example, the *tmk* genes from the JWB phytoplasma, highlighted with green triangles, red squares, and purple circles in Figure 5, were clustered into subclades C and D. In addition, the *tmk* genes of the PaWB (paulownia witches’ broom) and JWB phytoplasmas from China belonging to the 16SrI and V groups, respectively, were clustered together as the C-1 subclade, where the *tmk8* of PaWB-Zhengzhou was sandwiched between several *tmk* genes from JWB phytoplasmas. The *tmk8* gene of PaWB-Zhengzhou from Henan Province shared a 99.84% identity (one different base) with the *tmk2* gene of JWB-nky from Shandong Province or the *tmk2* of JWB-Hebei-2018 from Hebei Province, and a 99.69% (two different bases) identity with the *tmk-x* gene of the JWB phytoplasmas from this study. Moreover, in our previous research, a *tmk* gene cloned from the Pingshan strain of the PaWB phytoplasma was identical to the *tmk-y* gene of the JWB phytoplasma [14]. However, PaWB-Zhengzhou shared a 89.90% and 89.29% identity in the 16Sr RNA gene with the JWB-nky and JWB-Hebei-2018 phytoplasmas, respectively (Figure S3). This evidence strongly demonstrates that recent HGT (horizontal gene transfer) events across genomes between the JWB and PaWB phytoplasmas happened in China.

## 4. Discussion

Genetic variations in the *tmk* genes of several phytoplasma strains, such as OY (onion yellow), WBD (wheat blue dwarf), and PaWB, were analyzed in previous studies [13,14,15]. We revealed the variations of *tmk* gene sequences in the JWB phytoplasma in this study, which significantly differed from conservative genes reported previously, such as 16Sr RNA, *rp*, *tuf*, and so on [26]. The *tmk* genes of the phytoplasmas were classified into two major categories (phylo-S and -M) or five groups (A, B, C, D, and E), based on their ancestor clade or subclade. Group A belonged to the phylo-S category. The phylo-M category included groups B, C, D, and E. Two *tmk* genes identified in the JWB phytoplasmas in this study belonged to group C of the phylo-M category. However, we were unable to obtain *tmk* belonging to the phylo-S category from different JWB strains. The screening results in this study indicate that all the phytoplasmas contained phylo-M *tmk*, and only a few phytoplasmas might not have contained phylo-S *tmk*. Two complete genomes, rather than contigs, of JWB phytoplasmas have been published in GenBank [11,12]. Based on the phylogenetic tree (Figure 5), the JWB-nky genome only contains phylo-M *tmk*, while JWB-Hebei-2018 contains both phlo-M and phlo-S *tmk* genes. To our knowledge, this is the first report on the variation and diversity of *tmk* genes in JWB phytoplasmas, as well as a comprehensive classification of *tmk* genes in phytoplasmas worldwide.

Most phytoplasmas whose genomes were sequenced contained more than two copies of the *tmk* genes (Table S2). For example, the strawberry lethal yellow phytoplasma (CPA str. NZSb11) of ‘*Candidatus* Phytoplasma australiense’ had 42 copies of the *tmk* gene, 26 of which differed from each other [27]. The multi-copy of the *tmk* gene is common in phytoplasmas, which is an important reason for the rich diversity of *tmk* genes. The type-XY JWB phytoplasma strain contained *tmk-x* and *tmk-y* genes. We could not determine whether the *tmk-x* and *tmk-y* genes existed in the same phytoplasma cell. Therefore, the *tmk* gene was multi-copied in a phytoplasma cell, and there may also be mixtures of phytoplasma cells with different genomic information, which might be another reason for the diversity of the *tmk* gene in a phytoplasma strain.

In this study, the phylogenetic tree (Figure 5) revealed the two different evolutionary pathways of the *tmk* genes of phytoplasmas. Single-copy *tmk* genes in the ancestor clade S evolved similarly to the 16S rRNA gene. However, the multi-copy of *tmk* genes frequently occurred in many phytoplasmas in the ancestor clade M, which might be associated with potential mobile units (PMUs). A PMU, a unique characteristic of phytoplasma genomes, is a cluster containing many genes, such as *fliA*, *ssb*, *dam*, *himA*, *hflB*, *smc*, *tmk*, *dnaB*, *dnaG*, *tra5*, and even effector genes [8,9]. It has been reported that PMUs duplicated within genomes result in multiple copies of these genes [8,9]. The JWB-nky phytoplasma complete genome, for instance, has four PMUs, and each PMU contains a *tmk* gene [12]. Thus, the multi-copy of *tmk* genes found in this study indicates that PMUs may occur in many JWB phytoplasma strains.

Apart from replicating and moving within the genome [8,9], fewer studies have shown that PMUs could be transferred across genomes when carrying *tmk* and other genes [28]. A study suggested that genes encoding effectors might have been brought into the PnWB phytoplasma and *Ca*. P. mali via PMU-mediated HGT [28]. In this study, we found that *tmk* genes had been shuttled between the JWB and PaWB phytoplasma genomes. This shuttle was highly likely to depend on PMU-mediated HGT due to *tmk’s* location in the PMU. HGT between organisms requires a shared ecological niche, such as an identical plant host or insect vector [29]. Some studies have indicated that the ecological niches shared by JWB and PaWB phytoplasmas exist in China. First, jujube and paulownia trees are widely distributed in China and sometimes live closely together [26,30,31]. Vector insects can feed on the two trees. Furthermore, the occurrence of JWB disease is sometimes related to the planting of paulownia trees around jujube trees [31]. Second, a study reported that paulownia trees showed symptoms of witches’ broom after being fed on by *Hishimonus chinesis* infected with JWB phytoplasmas [32]. Third, co-infection by JWB and PaWB phytoplasmas in jujube trees has been confirmed [33,34]. Perhaps the co-existence of JWB and PaWB phytoplasmas in the same hosts allows PMUs carrying *tmk* genes to shuttle between the two phytoplasma genomes.

Bacteria are ubiquitous on Earth and can inhabit almost every environment. They undergo many variations at the molecular level, such as mutation, recombination, transposition, and horizontal gene transfer, adapting to complex and ever-changing environments [4]. Phytoplasma resides exclusively in the sieve tubes of host plants to continue their life cycle [2]. Therefore, the disease resistance of plant hosts is a crucial environmental factor directly faced by phytoplasmas. Based on adaptive evolution by natural selection, beneficial variations that adapt to the environment are preserved, leading to a large population size. Conversely, harmful variations are gradually eliminated by the environment, resulting in a small size in their population. This study found that the type-X and type-XY phytoplasma strains accounted for a large proportion in nature and could infect resistant and susceptible jujube cultivars (Figure 4b,c). However, type-Y strains were rare and only found in susceptible cultivars (Figure 4c). Therefore, it could be seen that type-X and type-XY phytoplasma strains containing the *tmk-x* only or *tmk-x* and *tmk-y* genes may have a relatively stronger adaptability to jujube trees than the type-Y strain containing the *tmk-y* gene only.

Based on the relationships between the *tmk* genes of phytoplasmas and the resistant levels of hosts, putative phytoplasma resistant plants could be quickly screened by developing an artificially infecting platform. After being infected by the phytoplasmas and cultivated for a period of time, the potential resistance of the test plants may be preliminarily determined through detecting the genes of *tmk-x* and *tmk-y.* Furthermore, *tmk* mutations might increase the host adaptability of phytoplasma alone or in combination with other genes, which should be further determined in a future study. Clarifying the function and interaction mechanism of *tmk*, reducing its toxicity or adaptability through genetic engineering editing, and increasing the genetic diversity of the cultivated crops would be beneficial for preventing and controlling the loss of plant disease resistance in agricultural production.

## 5. Conclusions

The following conclusions were obtained in this study: (1) In terms of evolutionary patterns, phytoplasmas contain two major categories of *tmk* genes, phylo-S and phylo-M *tmk*. Phylo-S is single-copied in the phytoplasma genome and has a similar evolutionary history to the 16S rRNA gene, while phylo-M *tmk* is multi-copied, and its evolution is related to PMU-mediated HGT. (2) There are three types of JWB phytoplasma strains, namely, type-X, type-Y, and type-XY. Type-X and type-XY are dominant JWB phytoplasma strains in China, but type-Y is a subordinate strain. (3) The *tmk* gene belonging to the phylo-M category is shuttled between JWB and PaWB phytoplasmas in China. Revealing the relationship between the variations of the functional genes of phytoplasmas and the disease-resistant levels of the host plants allows for the rapid screening and identification of phytoplasma-resistant plant resources. Furthermore, these findings offer insights for further understanding genetic variations and the mechanisms of the adaptive evolution of phytoplasmas.

## Data Availability

Data are contained within the article or Supplementary Materials.

## Supplementary Materials

The following supporting information can be downloaded at: https://www.mdpi.com/article/10.3390/biology13110886/s1, Table S1. The JWB phytoplasma strains used in this study and experiments conducted to determine the types of strains; Table S2. Information of *tmk* genes employed for phylogenetic tree in this study; Table S3. The maximum, minimum, and average distances within the ancestor clade S and M of the *tmk* sequences of phytoplasmas; Figure S1. Sequencing chromatograms of the 14 regular nucleotide variation sites of the *tmk* genes of phytoplasma strains of type-X, type-Y and type-XY; Figure S2. The phylogenetic tree respectively based on phylo-S *tmk* and 16S rRNA gene from phytoplasmas; Figure S3. Phytoplasma strain PaWB-Zhengzhou shared 89.90% and 89.29% identity in 16S rRNA gene respectively with JWB-nky and JWB-Hebei-2018 phytoplasma.
