# Supplementary material for: Molecular Variation and Phylogeny of Thymidylate Kinase Genes of Candidatus Phytoplasma ziziphi from Different Resistant and Susceptible Jujube Cultivars in China
Source: Biology (Basel). 2024 Oct 30;13(11):886. doi: 10.3390/biology13110886 (PMC11592322; doi:10.3390/biology13110886)
Supplement: Supplementary file 1 [file biology-13-00886-s001.zip › biology-3247940-supplementary.pdf]

Supplementary Materials

Table S1. The JWB phytoplasma strains used in this study and experiments conducted to determine the types of strains

JWB phytoplasma strains	Infected cultivars	Provinces	Sampling sites	Disease resistances	Cloned-based Sequencing	Direct sequencing	Selective PCR	Types of JWB phytoplasma strains
BJLZ	Longzao	Beijing	Xuanwu district	S	+	+	+	Type-X
BLDZ	Dongzao	Beijing	Changping district	S		+		Type-XY
BSDA	Beishi Dazao	Beijing	Xuanwu district	U	+	+		Type-XY
BSSZ-a	Suanzao	Shandong	Boshan district	S		+		Type-X
BSSZ-b	Suanzao	Shandong	Boshan district	S		+		Type-X
BSSZ-c	Suanzao	Shandong	Boshan district	S		+		Type-X
CAFL	Lizao	Beijing	Haidian district	S		+		Type-X
CP126	Changping 126zao	Beijing	Changping district	R		+		Type-X
CPLZ	Lizao	Beijing	Changping district	S		+	+	Type-XY
CPMY	Mayazao	Beijing	Changping district	U	+	+	+	Type-XY
CPYG	Yueguangzao	Beijing	Changping district	S		+		Type-XY
FPHP	Hupingzao	Hebei	Fuping county	R		+		Type-XY
FPPZ	Pozao	Hebei	Fuping county	S	+	+		Type-Y
HDDM	Dongzao	Beijing	Haidian district	S		+		Type-XY
HDDO	Dongzao	Beijing	Haidian district	S		+	+	Type-X
JADA	Ji'an Dazao	Jiangxi	Ji'an city	U	+	+	+	Type-XY
JSBZ	Banzao	Shanxi	Jishan county	R		+		Type-X
JSSZ	Suanzao	Shanxi	Jishan county	S		+		Type-XY
NYCH-a	Changhongzao	Shandong	Ningyang county	R	+	+	+	Type-X

JWB phytoplasma strains	Infected cultivars	Provinces	Sampling sites	Disease resistances	Cloned-based Sequencing	Direct sequencing	Selective PCR	Types of JWB phytoplasma strains
NYCH-b	Changhongzao	Shandong	Ningyang county	R		+		Type-X
NYCH-c	Changhongzao	Shandong	Ningyang county	R		+		Type-X
NYYH-a	Yanhongzao	Shandong	Ningyang county	S	+	+	+	Type-X
NYYH-b	Yanhongzao	Shandong	Ningyang county	S		+		Type-X
NYYH-c	Yanhongzao	Shandong	Ningyang county	S		+		Type-X
PBHS	Bianhe Suanzao	Henan	Puyang city	S		+		Type-XY
PGDO	Dongzao	Beijing	Pinggu district	S	+	+		Type-X
PGLZ	Lizao	Beijing	Pinggu district	S	+	+		Type-X
PSHP	Hupingzao	Hebei	Pingshan county	R		+		Type-XY
SHDZ	Sihong Dazao	Jiangsu	Sihong county	S	+	+		Type-XY
TACZ	Cuizao	Shandong	Tai'an city	S	+	+		Type-X
TBDZ	Tongbai Dazao	Henan	Nanyang city	S		+		Type-Y
TJDO	Dongzao	Tianjin	Hexi district	S	+	+	+	Type-X
TXCH	Changhongzao	Hebei	Tang county	R		+	+	Type-XY
TXDO	Dongzao	Hebei	Tang county	S	+	+	+	Type-XY
TXPZ-a	Pozao	Hebei	Tang county	S		+	+	Type-Y
TXPZ-b	Pozao	Hebei	Tang county	S	+	+		Type-XY
TXPZ-c	Pozao	Hebei	Tang county	S		+		Type-Y
TXSZ-a	Suanzao	Hebei	Tang county	S		+	+	Type-XY
TXSZ-b	Suanzao	Hebei	Tang county	S	+	+		Type-Y
XFDO	Dongzao	Beijing	Changping district	S		+	+	Type-XY
XFXZ	Xifengshan Xiaozao	Beijing	Changping district	U	+	+	+	Type-XY

**JWB phytoplasma strains**	**Infected cultivars**	**Provinces**	**Sampling sites**	**Disease resistances**	**Cloned-based Sequencing**	**Direct sequencing**	**Selective PCR**	**Types of JWB phytoplasma strains**
XFYZ	Yuanzao	Shanxi	Xiangfen county	S		+		Type-X
XTLZ	Lizao	Hebei	Xingtang county	S		+		Type-Y
XTPZ	Pozao	Hebei	Xingtang county	S		+		Type-XY
XYPZ	Pozao	Hebei	Tang county	S		+	+	Type-XY
YCLZ	Lizao	Shanxi	Yuncheng city	S		+		Type-XY
YCXZ	Xiangzao	Shanxi	Yuncheng city	S		+		Type-XY
ZHDA	Zanhuang Dazao	Hebei	Zanhuang county	S		+		Type-X
ZHSZ	Suanzao	Hebei	Zanhuang county	S		+		Type-X
ZSSZ	Suanzao	Henan	Luoyang city	S		+		Type-XY

S and R respectively represented the 'Susceptible' and 'Resistant' to JWB disease, U represented unknown. '+' represented the experiment was conducted.

Table S2. Information of *tmk* genes employed for phylogenetic tree in the study

**Leaves name of phylogenetic tree**	**Phytoplasma name abbreviation**	**Strain**	**16Sr group**	**Gene name**	**Accession number**	**Locus tag**	**Ancestor clade in tmk phylogenetic tree of Figure 7**
AYWB *tmk1*	AYWB		16Sr I	*tmk1*	CP000061.1	AYWB_074	M
AYWB *tmk2*	AYWB		16Sr I	*tmk2*	CP000061.1	AYWB_181	M
AYWB *tmk3*	AYWB		16Sr I	*tmk3*	CP000061.1	AYWB_222	M
AYWB *tmk4*	AYWB		16Sr I	*tmk4*	CP000061.1	AYWB_282	M
AYWB *tmk5*	AYWB		16Sr I	*tmk5*	CP000061.1	AYWB_492	S
OY-M *tmk1*	OY	OY-M	16Sr I	*tmk1*	AP006628.2	PAM_230	S
OY-M *tmk2*	OY	OY-M	16Sr I	*tmk2*	AP006628.2	PAM_344	M
OY-M *tmk4*	OY	OY-M	16Sr I	*tmk4*	AP006628.2	PAM_521	M

**Leaves name of phylogenetic tree**	**Phytoplasma name abbreviation**	**Strain**	**16Sr group**	**Gene name**	**Accession number**	**Locus tag**	**Ancestor clade in tmk phylogenetic tree of Figure 7**
OY-M *tmk5*	OY	OY-M	16Sr I	*tmk5*	AP006628.2	PAM_688	M
MBS M3 *tmk*	MBS	M3	16Sr I	*tmk*	CP015149.1	MBSPM3_v1c3530	S
BN TW1 *tmk1*	BN	TW1	16Sr I	*tmk1*	QGKT01000001.1	DF280_00335	S
BN TW1 *tmk2*	BN	TW1	16Sr I	*tmk2*	QGKT01000001.1	DF280_01635	M
BN TW1 *tmk3*	BN	TW1	16Sr I	*tmk3*	QGKT01000002.1	DF280_02735	M
WBD *tmk1*	WBD	WBD	16Sr I	*tmk1*	NZ_AVAO01000003.1	N747_RS0101490	M
WBD *tmk2*	WBD	WBD	16Sr I	*tmk2*	NZ_AVAO01000003.1	N747_RS0102340	M
WBD *tmk3*	WBD	WBD	16Sr I	*tmk3*	NZ_AVAO01000003.1	N747_RS0102615	S
MDGZ-01 *tmk*	MD	MDGZ-01	16Sr I	*tmk*	NZ_CP085837.1	LJP71_RS01255	S
EAWB TBZ1 *tmk1*	EAWB	TBZ1	16Sr I	*tmk1*	JAHFWK010000005	KEC49_00760	S
EAWB TBZ1 *tmk2*	EAWB	TBZ1	16Sr I	*tmk2*	JAHFWK010000021	KEC49_01705	M
HP *tmk1*	HP	HP	16Sr I	*tmk1*	NZ_BMZZ01000005.1	KI197_RS01725	S
SAAY SW86 *tmk1*	SAAY	SW86	16Sr I	*tmk1*	NZ_JAGVSK010000001.1	KE631_RS00115	S
SAAY SW86 *tmk2*	SAAY	SW86	16Sr I	*tmk2*	NZ_JAGVSK010000001.1	KE631_RS00330	M
PLY DY2014 *tmk1*	PLY	DY2014	16Sr I	*tmk1*	SRMC01000002.1	PLY_1070	M
PLY DY2014 *tmk2*	PLY	DY2014	16Sr I	*tmk2*	SRMC01000002.1	PLY_1240	M
PLY DY2014 *tmk5*	PLY	DY2014	16Sr I	*tmk5*	SRMC01000005.1	PLY_3910	M
PLY DY2014 *tmk6*	PLY	DY2014	16Sr I	*tmk6*	SRMC01000005.1	PLY_4170	M
PLY DY2014 *tmk7*	PLY	DY2014	16Sr I	*tmk7*	SRMC01000006.1	PLY_5020	M
PLY DY2014 *tmk8*	PLY	DY2014	16Sr I	*tmk8*	SRMC01000007.1	PLY_5620	M
PLY DY2014 *tmk9*	PLY	DY2014	16Sr I	*tmk9*	SRMC01000008.1	PLY_7030	S
RP166 *tmk1*	RP	RP166	16Sr I	*tmk1*	CP055264.1	RP166_0870	M
RP166 *tmk2*	RP	RP166	16Sr I	*tmk2*	CP055264.1	RP166_1600	M

**Leaves name of phylogenetic tree**	**Phytoplasma name abbreviation**	**Strain**	**16Sr group**	**Gene name**	**Accession number**	**Locus tag**	**Ancestor clade in tmk phylogenetic tree of Figure 7**
RP166 *tmk4*	RP	RP166	16Sr I	*tmk4*	CP055264.1	RP166_2240	M
RP166 *tmk5*	RP	RP166	16Sr I	*tmk5*	CP055264.2	RP166_2410	M
RP166 *tmk6*	RP	RP166	16Sr I	*tmk6*	CP055264.1	RP166_2610	M
RP166 *tmk7*	RP	RP166	16Sr I	*tmk7*	CP055264.1	RP166_3320	M
RP166 *tmk8*	RP	RP166	16Sr I	*tmk8*	CP055264.1	RP166_4170	S
RP166 *tmk9*	RP	RP166	16Sr I	*tmk9*	CP055264.1	RP166_5110	M
RP166 *tmk10*	RP	RP166	16Sr I	*tmk10*	CP055264.1	RP166_5350	M
RP166 *tmk12*	RP	RP166	16Sr I	*tmk12*	CP055264.1	RP166_7140	M
RP166 *tmk13*	RP	RP166	16Sr I	*tmk13*	CP055264.1	RP166_8280	M
CRAY De Villa *tmk*	CRAY	De Villa	16Sr I	*tmk*	NZ_CP035949.1	EXT02_RS01875	S
NJAY *tmk1*	NJAY	NJAY	16Sr I	*tmk1*	NZ_MAPF01000014.1	BBA70_RS00420	M
NJAY *tmk2*	NJAY	NJAY	16Sr I	*tmk2*	NZ_MAPF01000026.1	BBA70_RS00950	M
NJAY *tmk3*	NJAY	NJAY	16Sr I	*tmk3*	NZ_MAPF01000026.1	BBA70_RS01145	M
NJAY *tmk4*	NJAY	NJAY	16Sr I	*tmk4*	NZ_MAPF01000056.1	BBA70_RS02595	S
ROLP LD1 *tmk1*	ROLP	LD1	16Sr I	*tmk1*	NZ_MIEP01000004.1	BHE82_RS01580	S
CYP *tmk1*	CYP	CYP	16Sr I	*tmk1*	NZ_JSWH01000098.1	OL66_RS01930	M
CYP *tmk2*	CYP	CYP	16Sr I	*tmk2*	NZ_JSWH01000107.1	OL66_RS02235	S
CYP *tmk3*	CYP	CYP	16Sr I	*tmk3*	NZ_JSWH01000119.1	OL66_RS02455	M
ChC OY-V *tmk1*	ChC	OY-V	16Sr I	*tmk1*	NZ_BBIY01000021.1	OYV_RS01165	M
ChC OY-V *tmk2*	ChC	OY-V	16Sr I	*tmk2*	NZ_BBIY01000027.1	OYV_RS01560	M
ChC OY-V *tmk3*	ChC	OY-V	16Sr I	*tmk3*	NZ_BBIY01000028.1	OYV_RS01680	M
ChC OY-V *tmk4*	ChC	OY-V	16Sr I	*tmk4*	NZ_BBIY01000031.1	OYV_RS01975	S
ChC OY-V *tmk5*	ChC	OY-V	16Sr I	*tmk5*	NZ_BBIY01000040.1	OYV_RS02565	M

**Leaves name of phylogenetic tree**	**Phytoplasma name abbreviation**	**Strain**	**16Sr group**	**Gene name**	**Accession number**	**Locus tag**	**Ancestor clade in tmk phylogenetic tree of Figure 7**
ChC OY-V *tmk6*	ChC	OY-V	16Sr I	*tmk6*	NZ_BBIY01000056.1	OYV_RS03080	M
ChC OY-V *tmk7*	ChC	OY-V	16Sr I	*tmk7*	NZ_BBIY01000061.1	OYV_RS03180	M
ChC OY-V *tmk8*	ChC	OY-V	16Sr I	*tmk8*	NZ_BBIY01000065.1	OYV_RS03285	M
PaWB zhengzhou *tmk1*	PaWB	zhengzhou	16Sr I	*tmk1*	CP066882.1	HGD80_00105	S
PaWB zhengzhou *tmk2*	PaWB	zhengzhou	16Sr I	*tmk2*	CP066882.1	HGD80_00895	M
PaWB zhengzhou tmk4	PaWB	zhengzhou	16Sr I	*tmk4*	CP066882.1	HGD80_01085	M
PaWB zhengzhou *tmk7*	PaWB	zhengzhou	16Sr I	*tmk7*	CP066882.1	HGD80_02620	M
PaWB zhengzhou *tmk8*	PaWB	zhengzhou	16Sr I	*tmk8*	CP066882.1	HGD80_02750	M
PaWB zhengzhou *tmk9*	PaWB	zhengzhou	16Sr I	*tmk9*	CP066882.1	HGD80_02880	M
PaWB JAN *tmk-b*	PaWB	JAN	16Sr I	*tmk-b*	KJ452544.1		S
PaWB PS *tmk-b*	PaWB	PS	16Sr I	*tmk-b*	KJ452545.1		S
PaWB JAN *tmk-a-1*	PaWB	JAN	16Sr I	*tmk-a-1*	KJ452468.1		M
PaWB JAN *tmk-a-2*	PaWB	JAN	16Sr I	*tmk-a-2*	KJ452469.1		M
PaWB PS *tmk-a-1*	PaWB	PS	16Sr I	*tmk-a-1*	KJ452507.1		M
PaWB PS *tmk-a-2*	PaWB	PS	16Sr I	*tmk-a-2*	KJ452492.1		M
PspP PR08 *tmk*	PspP	PR08	16Sr II	*tmk*	NZ_JAGXLX010000001.1		S
PHP PR34 *tmk*	PHP	PR34	16Sr II	*tmk*	NZ_JACRYS020000007.1	H7686_RS01770	S
WBDL *tmk*	WBDL	WBDL	16Sr II	*tmk*	NZ_MWKN01000015.1	B2G44_RS00580	S
EPWB NCHU2014 *tmk1*	EPWB	NCHU2014	16Sr II	*tmk1*	CP040925.1	EPWB_v2c1820	S
EPWB NCHU2014 *tmk2*	EPWB	NCHU2014	16Sr II	*tmk2*	CP040925.1	EPWB_v2c2590	M
PNWB NUT2011 *tmk*	PNWB	NUT2011	16Sr II	*tmk*	NZ_AMWZ01000013.1	PNWB_RS02430	S
SeP SS02 *tmk*	SeP	SS02	16Sr II	*tmk*	NZ_JAHBAJ020000012.1	KHD59_RS01690	S
VAC *tmk*	VAC	VAC	16Sr III	*tmk*	NZ_AKIN01000059.1	RI68_RS01660	S

**Leaves name of phylogenetic tree**	**Phytoplasma name abbreviation**	**Strain**	**16Sr group**	**Gene name**	**Accession number**	**Locus tag**	**Ancestor clade in tmk phylogenetic tree of Figure 7**
ICPH MA1 *tmk1*	ICPh	MA1	16Sr III	*tmk1*	NZ_AKIM01000051.1	RI01_RS02255	M
ICPH MA1 *tmk3*	ICPh	MA1	16Sr III	*tmk3*	NZ_AKIM01000007.1	RI01_RS00230	S
MiY MW1 *tmk*	MiY	MW1	16Sr III	*tmk*	NZ_AKIL01000039.1	RI76_RS00770	S
PoiBI JR1 *tmk*	PoiBI	JR1	16Sr III	*tmk*	NZ_AKIK01000045.1	ON17_RS01280	S
ChTDIII *tmk1*	ChTD	ChTDIII	16Sr III	*tmk1*	NZ_JABUOH010000041.1	HR065_RS01515	M
ChTDIII *tmk2*	ChTD	ChTDIII	16Sr III	*tmk2*	NZ_JABUOH010000047.1	HR065_RS01900	S
CX *tmk1*	CX	CX	16Sr III	*tmk1*	NZ_LHCF01000006.1	CPX_RS01325	S
CX *tmk2*	CX	CX	16Sr III	*tmk2*	NZ_LHCF01000015.1	CPX_RS02200	M
ZT3-1 *tmk*		ZT3-1	16Sr III	*tmk*	JANIEM010000003	NOI47_000268	S
TePP ACPD *tmk2*	TePP	ACPD	16Sr IV	*tmk2*	NZ_VBRA02000009.1	FEF22_RS01160	S
JWB-nky *tmk1*	JWB	JWB-nky	16Sr V	*tmk1*	CP025121.1	CWO85_00825	M
JWB-nky *tmk2*	JWB	JWB-nky	16Sr V	*tmk2*	CP025121.1	CWO85_00915	M
JWB Hebei 2018 *tmk1*	JWB	Hebei-2018	16Sr V	*tmk1*	NZ_CP091835.1	L6W10_RS00445	M
JWB Hebei 2018 *tmk2*	JWB	Hebei-2018	16Sr V	*tmk2*	NZ_CP091835.1	L6W10_RS00960	M
JWB Hebei 2018 *tmk3*	JWB	Hebei-2018	16Sr V	*tmk3*	NZ_CP091835.1	L6W10_RS02290	M
JWB Hebei 2018 *tmk4*	JWB	Hebei-2018	16Sr V	*tmk4*	NZ_CP091835.1	L6W10_RS03115	S
CH *tmk*		CH	16Sr V	*tmk*	CP097583.1	M6G77_01710	S
LFWB NCHU2019 *tmk1*	LFWB	NCHU2019	16Sr VIII	*tmk1*	NZ_CP054393.1	LFWB_RS00200	M
LFWB NCHU2019 *tmk4*	LFWB	NCHU2019	16Sr VIII	*tmk4*	NZ_CP054393.1	LFWB_RS01200	S
LFWB *tmk*	LFWB		16Sr VIII	*tmk*	AF251151.1		M
PP2 *tmk1*	PP	PP2	16Sr IX	*tmk1*	JAIFKF010000001	J8272_00015	M
AlmWB SA213 *tmk1*	AlmWB	SA213	16Sr IX	*tmk1*	NZ_JPSQ01000001.1	AlmWB_RS01770	M
AlmWB SA213 *tmk2*	AlmWB	SA213	16Sr IX	*tmk2*	NZ_JPSQ01000002.1	AlmWB_RS00060	S

**Leaves name of phylogenetic tree**	**Phytoplasma name abbreviation**	**Strain**	**16Sr group**	**Gene name**	**Accession number**	**Locus tag**	**Ancestor clade in tmk phylogenetic tree of Figure 7**
ChiP *tmk1*	ChiP	ChiP	16Sr IX	*tmk1*	PUUG01000005.1	C6B37_00410	S
ChiP *tmk2*	ChiP	ChiP	16Sr IX	*tmk2*	PUUG01000035.1	C6B37_01380	M
ChiP *tmk3*	ChiP	ChiP	16Sr IX	*tmk3*	PUUG01000051.1	C6B37_01735	M
ChiP *tmk4*	ChiP	ChiP	16Sr IX	*tmk4*	PUUG01000053.1	C6B37_01815	M
AP AT *tmk1*	AP	AT	16Sr X	*tmk1*	CU469464.1	ATP_0037	S
AP AT *tmk2*	AP	AT	16Sr X	*tmk2*	CU469464.1	ATP_00403	M
NGS-S10 *tmk1*	NGS	NGS-S10	16Sr XI	*tmk1*	NZ_JHUK01000003.1	DH96_RS01270	S
NGS-S10 *tmk2*	NGS	NGS-S10	16Sr XI	*tmk2*	NZ_JHUK01000004.1	DH96_RS01670	M
NGS-S10 *tmk3*	NGS	NGS-S10	16Sr XI	*tmk3*	NZ_JHUK01000004.1	DH96_RS01780	M
NGS Mbital *tmk1*	NGS	Mbita1	16Sr XI	*tmk1*	NZ_LTBM01000012.1	AXA84_RS01680	S
NGS Mbital *tmk2*	NGS	Mbita1	16Sr XI	*tmk2*	NZ_LTBM01000006.1	AXA84_RS01210	M
SCGS *tmk*	SCGS	SCGS	16Sr XI	*tmk*	NZ_VWXM01000002.1	F2B49_RS00420	S
CyD LW01 *tmk*	CyD	LW01	16Sr XI	*tmk*	NZ_VWOH01000001.1	F1741_RS00195	S
Tabriz2 *tmk2*		Tabriz.2	16Sr XII	*tmk2*	JAINCS010000090	K6L59_00905	M
Tabriz2 *tmk4*		Tabriz.2	16Sr XII	*tmk4*	JAINCS010000013	K6L59_00240	M
CPA NZSb11 *tmk1*	CPA	NZSb11	16Sr XII	*tmk1*	CP002548.1	SLY_0057	M
CPA NZSb11 *tmk2*	CPA	NZSb11	16Sr XII	*tmk2*	CP002548.1	SLY_0056	M
CPA NZSb11 *tmk3*	CPA	NZSb11	16Sr XII	*tmk3*	CP002548.1	SLY_0126	M
CPA NZSb11 *tmk4*	CPA	NZSb11	16Sr XII	*tmk4*	CP002548.1	SLY_0127	M
CPA NZSb11 *tmk5*	CPA	NZSb11	16Sr XII	*tmk5*	CP002548.1	SLY_0168	M
CPA NZSb11 *tmk6*	CPA	NZSb11	16Sr XII	*tmk6*	CP002548.1	SLY_0169	M
CPA NZSb11 *tmk7*	CPA	NZSb11	16Sr XII	*tmk7*	CP002548.1	SLY_0220	M
CPA NZSb11 *tmk8*	CPA	NZSb11	16Sr XII	*tmk8*	CP002548.1	SLY_0221	M

**Leaves name of phylogenetic tree**	**Phytoplasma name abbreviation**	**Strain**	**16Sr group**	**Gene name**	**Accession number**	**Locus tag**	**Ancestor clade in tmk phylogenetic tree of Figure 7**
CPA NZSb11 *tmk9*	CPA	NZSb11	16Sr XII	*tmk9*	CP002548.1	SLY_0249	M
CPA NZSb11 *tmk10*	CPA	NZSb11	16Sr XII	*tmk10*	CP002548.1	SLY_0465	M
CPA NZSb11 *tmk14*	CPA	NZSb11	16Sr XII	*tmk14*	CP002548.1	SLY_0629	M
CPA NZSb11 *tmk15*	CPA	NZSb11	16Sr XII	*tmk15*	CP002548.1	SLY_0630	M
CPA NZSb11 *tmk16*	CPA	NZSb11	16Sr XII	*tmk16*	CP002548.1	SLY_0642	M
CPA NZSb11 *tmk17*	CPA	NZSb11	16Sr XII	*tmk17*	CP002548.1	SLY_0643	M
CPA NZSb11 *tmk18*	CPA	NZSb11	16Sr XII	*tmk18*	CP002548.1	SLY_0691	M
CPA NZSb11 *tmk24*	CPA	NZSb11	16Sr XII	*tmk24*	CP002548.1	SLY_0832	M
CPA NZSb11 *tmk26*	CPA	NZSb11	16Sr XII	*tmk26*	CP002548.1	SLY_0852	M
CPA NZSb11 *tmk27*	CPA	NZSb11	16Sr XII	*tmk27*	CP002548.1	SLY_0853	M
CPA NZSb11 *tmk28*	CPA	NZSb11	16Sr XII	*tmk28*	CP002548.1	SLY_0918	S
CPA NZSb11 *tmk29*	CPA	NZSb11	16Sr XII	*tmk29*	CP002548.1	SLY_0946	M
CPA NZSb11 *tmk30*	CPA	NZSb11	16Sr XII	*tmk30*	CP002548.1	SLY_0947	M
CPA NZSb11 *tmk33*	CPA	NZSb11	16Sr XII	*tmk33*	CP002548.1	SLY_0983	M
CPA NZSb11 *tmk34*	CPA	NZSb11	16Sr XII	*tmk34*	CP002548.1	SLY_0984	M
CPA NZSb11 *tmk35*	CPA	NZSb11	16Sr XII	*tmk35*	CP002548.1	SLY_1023	M
CPA NZSb11 *tmk36*	CPA	NZSb11	16Sr XII	*tmk36*	CP002548.1	SLY_1024	M
CPA NZSb11 *tmk38*	CPA	NZSb11	16Sr XII	*tmk38*	CP002548.1	SLY_1078	M
CPA NZSb11 *tmk39*	CPA	NZSb11	16Sr XII	*tmk39*	CP002548.1	SLY_1082	M
CPA NZSb11 *tmk40*	CPA	NZSb11	16Sr XII	*tmk40*	CP002548.1	SLY_1083	M
CPA NZSb11 *tmk42*	CPA	NZSb11	16Sr XII	*tmk42*	CP002548.1	SLY_1086	M
AM422018 *tmk3*			16Sr XII	*tmk3*	AM422018.1	PA0169	S
AM422018 *tmk4*			16Sr XII	*tmk4*	AM422018.1	PA0195	M

**Leaves name of phylogenetic tree**	**Phytoplasma name abbreviation**	**Strain**	**16Sr group**	**Gene name**	**Accession number**	**Locus tag**	**Ancestor clade in tmk phylogenetic tree of Figure 7**
AM422018 *tmk6*			16Sr XII	*tmk6*	AM422018.1	PA0304	M
AM422018 *tmk9*			16Sr XII	*tmk9*	AM422018.1	PA0373	M
AM422018 *tmk10*			16Sr XII	*tmk10*	AM422018.1	PA0425	M
Stolbur 284-09 *tmk1*	Stolbur	284-09	16Sr XII	*tmk1*	FO393427.1	S284_00590	M
Stolbur 284-09 *tmk3*	Stolbur	284-09	16Sr XII	*tmk3*	FO393427.1	S284_02410	S
Stolbur 239-01 *tmk2*	Stolbur	239-01	16Sr XII	*tmk2*	FO393428.1	S231_02230	S
Stolbur 239-01 *tmk3*	Stolbur	239-01	16Sr XII	*tmk3*	FO393428.1	S231_04630	M
Stolbur SA-1 *tmk1*	Stolbur	SA-1	16Sr XII	*tmk1*	NZ_MPBG01000001.1	PSSA1_RS00585	M
Stolbur SA-1 *tmk2*	Stolbur	SA-1	16Sr XII	*tmk2*	NZ_MPBG01000002.1	PSSA1_RS00885	S
Stolbur SA-1 *tmk3*	Stolbur	SA-1	16Sr XII	*tmk3*	NZ_MPBG01000013.1	PSSA1_RS03450	M
Stolbur SA-1 *tmk4*	Stolbur	SA-1	16Sr XII	*tmk4*	NZ_MPBG01000017.1	PSSA1_RS03760	M
FAP StrPh-Cl *tmk1*	FAP	StrPh-Cl	16Sr XIII	*tmk1*	NZ_JAGVRH010000021.1	J8J04_RS02935	M
FAP StrPh-Cl *tmk2*	FAP	StrPh-Cl	16Sr XIII	*tmk2*	NZ_JAGVRH010000003.1	J8J04_RS01275	S
FAP StrPh-Cl *tmk3*	FAP	StrPh-Cl	16Sr XIII	*tmk3*	NZ_JAGVRH010000005.1	J8J04_RS01810	M
ChTYXIII-Mo *tmk1*	ChTY	ChTYXIII-Mo	16Sr XIII	*tmk1*	NZ_JACAOD020000001.1	CHTY_RS00035	M
ChTYXIII-Mo *tmk2*	ChTY	ChTYXIII-Mo	16Sr XIII	*tmk2*	NZ_JACAOD020000001.1	CHTY_RS00250	S
ChTYXIII-Mo *tmk3*	ChTY	ChTYXIII-Mo	16Sr XIII	*tmk3*	NZ_JACAOD020000012.1	CHTY_RS02605	M
ChTYXIII-Mo *tmk4*	ChTY	ChTYXIII-Mo	16Sr XIII	*tmk4*	NZ_JACAOD020000003.1	CHTY_RS01090	M
ChTYXIII-Mo *tmk5*	ChTY	ChTYXIII-Mo	16Sr XIII	*tmk5*	NZ_JACAOD020000005.1	CHTY_RS01470	M
PTWB MDPP *tmk*	PTWB	MDPP	16Sr XXI	*tmk*	NZ_VIAE01000002.1	MDPP_RS00520	S
Escherichia coli tmk		Sakai		*tmk*	BA000007.3	ECs_1476	S
Xanthomonas campestris tmk		MAFF106181		*tmk*	CP058243.1	AD14011_19710	S
Chlamydia trachomatis tmk		D/UW-3/CX		*tmk*	AE001273.1	CT_188	S

**Leaves name of phylogenetic tree**	**Phytoplasma name abbreviation**	**Strain**	**16Sr group**	**Gene name**	**Accession number**	**Locus tag**	**Ancestor clade in tmk phylogenetic tree of Figure 7**
Chlamydia pecorum tmk		DBDeUG_2018		*tmk*	CP080401.1	DBDeUG_0768	S
Chlamydia muridarum tmk		Nigg		*tmk*	AE002160.2	TC_0460	S
Acholeplasma laidlawii tmk		PG-8A		*tmk*	NC_010163.1	ACL_0127	S
Acholeplasma granularum tmk		ATCC 19168		*tmk*	NZ_JAFR01000014.1	G324_RS0106740	S
Acholeplasma hippikon tmk		NCTC10172		*tmk*	LR215050.1	NCTC10172_00970	S
Spiroplasma citri tmk		R8-A2		*tmk*	CP013197.1	SCITRI_009	S
Spiroplasma platyhelix tmk		PALS-1		*tmk*	CP051215.1	SPLAT_v1c00150	S
Spiroplasma ixodetis tmk		DCF		*tmk*	JACSEQ010000001	H9Q65_00035	S
Mycoplasma capricolum tmk		ATCC 27343		*tmk*	CP000123.1	MCAP_0010	S
Mycoplasma mycoides tmk		GM12		*tmk*	CP001668.1	MMCAP2_0045	S
Mycoplasma nasistruthionis tmk		2F1A		*tmk*	CP040825.1	FG904_02790	S
Variola virus *tmk*		Pakistan 1969		*tmk*	DQ437589.1	VARV_PAK69_lah_165	S
Cowpox virus *tmk*		Ger/2010/Alpaca		*tmk*	LT896718.1	CPXV186	S
Vaccinia virus *tmk*		WR		*tmk*	AY243312.1	VACWR174	S
Arabidopsis thaliana ZEU1		Columbia		*ZEU1*	NM_125335	AT5G59440	S
Arabidopsis lyrata ZEU1		lyrata		*ZEU1*	XM_021011892.1	LOC9300711	S
Populus alba TMPK		PAL-ZL1		*TMPK*	XM_035050574.1	LOC118042850	S
Ziziphus jujuba TMPK		spinosa		*TMPK*	XM_048468292.1	LOC107434711	S
Mus musculus Dtymk		C57BL/6		*Dtymk*	NM_001105667.1		S
Homo sapiens DTYMK				*DTYMK*	NM_012145.4		S
JWB *tmk-x*	JWB			*tmk-x*	GU196274		M
JWB *tmk-y*	JWB			*tmk-y*	GU196275		M

Table S3. The maximum, minimum, and average distances within the ancestor clade S and M of the *tmk* sequences of phytoplasmas

	Maximum value	Minimum value	Average value
Ancestor Clade S	0.3960	0.0000	0.2819
Ancestor Clade M	0.6319	0.0000	0.3918

The multiple sequence alignment of the *tmk* sequences of phytoplasmas in ancestor clade S of Figure 7 was conducted, and then pairwise genetic distances (p-distance) was computed. The distance matrix was obtained. The multiple alignment and compute of the genetic distances were completed using MEGA 7.0 software. The maximum and minimum values in the matrix were filtered, and the average value was computed using Excel software. The parallel analyses of *tmk* sequences of phytoplasmas in ancestor clade M of Figure 7 were also conducted. The results indicated that the genetic distance range within clade S was narrower than clade M, and the average value of the genetic distance within clade S was smaller than clade M.

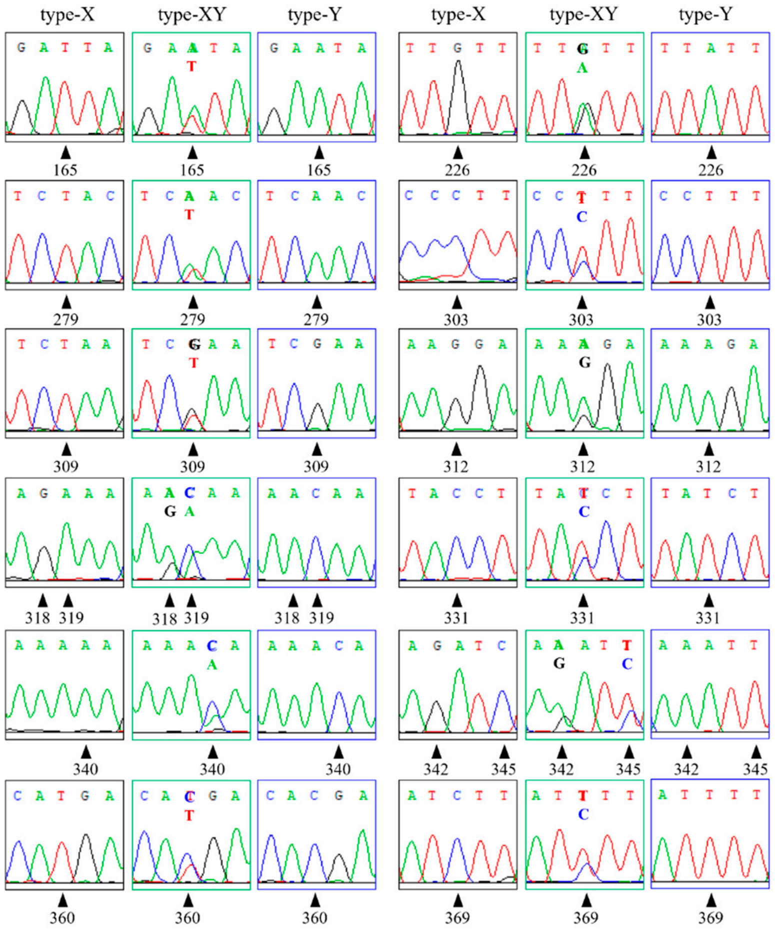

Figure S1. Sequencing chromatograms of the 14 regular nucleotide variation sites of the *tmk* genes of phytoplasma strains of type-X, type-XY and type-Y. Sequencing chromatograms of the type-X, type-XY and type-Y were highlighted with black, green and blue borders, respectively. Black triangles with numbers showed the variation sites.

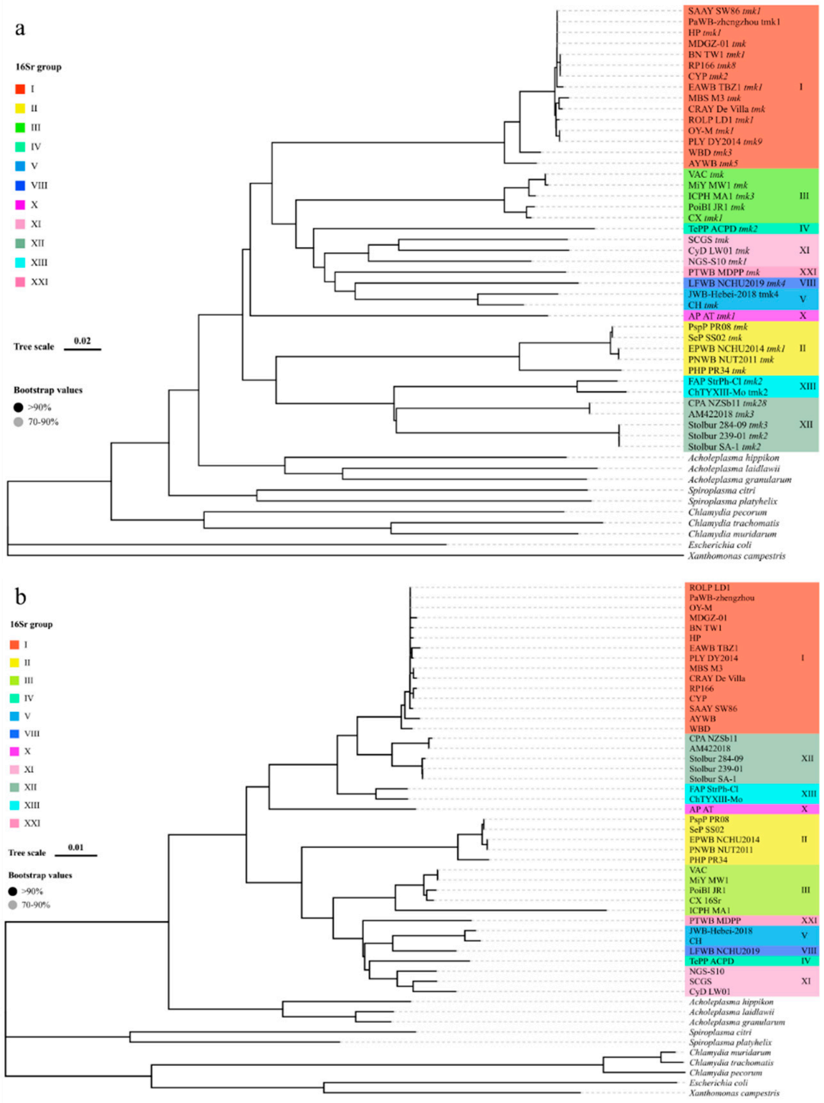

a
16Sr group
I
II
III
IV
V
VIII
X
XI
XII
XIII
XXI
Tree scale
0.02
Bootstrap values
>90%
70-90%
SAAY SW86 tmk1
PaWB-zhengzhou tmk1
HP tmk1
MDGZ-01 tmk
BN TW1 tmk1
RP166 tmk8
CYP tmk2
EAWB TBZ1 tmk1
MBS M3 tmk
CRAY De Villa tmk
ROLP LD1 tmk1
OY-M tmk1
PLY DY2014 tmk9
WBD tmk3
AYWB tmk5
VAC tmk
MiY MW1 tmk
ICPH MA1 tmk3
PoiBI JR1 tmk
CX tmk1
TePP ACPD tmk2
SCGS tmk
CyD LW01 tmk
NGS-S10 tmk1
PTWB MDPP tmk
LFWB NCHU2019 tmk4
JWB-Hebei-2018 tmk4
CH tmk
AP AT tmk1
PspP PR08 tmk
SeP SS02 tmk
EPWB NCHU2014 tmk1
PNWB NUT2011 tmk
PHP PR34 tmk
FAP StrPh-Cl tmk2
ChTYXIII-Mo tmk2
CPA NZSb11 tmk28
AM422018 tmk3
Stolbur 284-09 tmk3
Stolbur 239-01 tmk2
Stolbur SA-1 tmk2
Acholeplasma hippikon
Acholeplasma laidlawii
Acholeplasma granularum
Spiroplasma citri
Spiroplasma platyhelix
Chlamydia pecorum
Chlamydia trachomatis
Chlamydia muridarum
Escherichia coli
Xanthomonas campestris
b
16Sr group
I
II
III
IV
V
VIII
X
XI
XII
XIII
XXI
Tree scale
0.01
Bootstrap values
>90%
70-90%
ROLP LD1
PaWB-zhengzhou
OY-M
MDGZ-01
BN TW1
HP
EAWB TBZ1
PLY DY2014
MBS M3
CRAY De Villa
RP166
CYP
SAAY SW86
AYWB
WBD
CPA NZSb11
AM422018
Stolbur 284-09
Stolbur 239-01
Stolbur SA-1
FAP StrPh-Cl
ChTYXIII-Mo
AP AT
PspP PR08
SeP SS02
EPWB NCHU2014
PNWB NUT2011
PHP PR34
VAC
MiY MW1
PoiBI JR1
CX 16Sr
ICPH MA1
PTWB MDPP
JWB-Hebei-2018
CH
LFWB NCHU2019
TePP ACPD
NGS-S10
SCGS
CyD LW01
Acholeplasma hippikon
Acholeplasma laidlawii
Acholeplasma granularum
Spiroplasma citri
Spiroplasma platyhelix
Chlamydia muridarum
Chlamydia trachomatis
Chlamydia pecorum
Escherichia coli
Xanthomonas campestris

Figure S2. The phylogenetic tree respectively based on phylo-S *tmk* and 16S rRNA gene from phytoplasmas. (a) the phylo-S *tmk* phylogenetic tree. (b) the 16S rRNA phylogenetic tree. The sequence information used to construct two evolutionary trees were shown in Table S1. The evolutionary history was inferred using the Neighbor-Joining method. The percentages of replicate trees in which the associated taxa clustered together in the bootstrap test (1000 replicates) were shown next to the branches. Black circles indicated percentages of replicate trees greater than 90%, gray circles indicated percentages between 60-90%, and unmarked circles indicated percentages less than 60%.

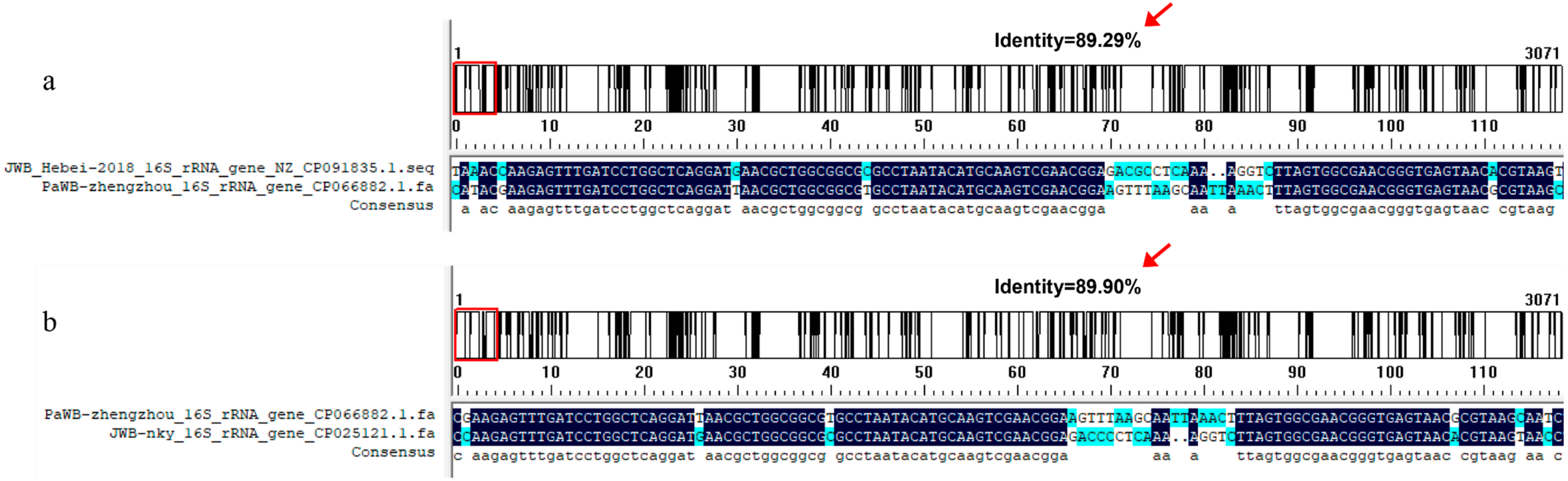

Figure S3. Phytoplasma strain PaWB-Zhengzhou shared 89.90% and 89.29% identity in 16Sr RNA gene respectively with JWB-nky and JWB-Hebei-2018 phytoplasma. (a) showed the identity between PaWB-Zhengzhou and JWB-Hebei-2018. (b) showed the identity between PaWB-Zhengzhou and JWB-nky. Pairwise comparisons were conducted and identity values were computed using DNAMAN 7.0 software. Identity values were highlighted with red arrows.
